# A qualitative study on sleep experiences in late pregnancy among women with a history of perinatal loss

**DOI:** 10.1371/journal.pone.0354895

**Published:** 2026-08-19

**Authors:** Yan Zhang, Jingge Liu, Ling Jiang, Xiuming Ji

**Affiliations:** Nursing Department, The Affiliated Suzhou Hospital of Nanjing Medical University, Suzhou Municipal Hospital, Suzhou, People’s Republic of China; Xiangya Hospital Central South University, CHINA

## Abstract

**Objective:**

To explore the sleep experiences in late pregnancy among women with a history of perinatal loss, aiming to provide a reference for developing relevant nursing programmes for this population.

**Methods:**

A descriptive qualitative study was conducted using purposeful sampling. Between October 2023 and February 2024, 18 late-trimester pregnant women with a history of perinatal loss were recruited from Suzhou Municipal Hospital for semi-structured interviews. Data were analysed and themes were extracted using Colaizzi’s seven-step analysis method.

**Results:**

Three main themes were identified: severe sleep disruption; diverse factors influencing sleep quality (including heavy psychological burden, pregnancy-related symptoms, behavioral and lifestyle factors, weak support from family and friends, and insufficient support from healthcare providers); and difficulties in self-managing sleep problems.

**Conclusion:**

Women with a history of perinatal loss often experience poor sleep quality during the third trimester of subsequent pregnancy. Healthcare providers should promptly identify patients’ physical discomfort symptoms, monitor their psychological well-being, address maladaptive cognitions and enhance sleep health awareness. Multidimensional support systems and interventions to improve self-coping capacity should be implemented to optimise sleep quality in these women.

## Introduction

Perinatal loss is defined in the psychosocial and nursing literature as an umbrella term encompassing any involuntary loss of a pregnancy or death of an infant from conception up to 28 days after birth, including miscarriage, stillbirth, and neonatal death [1,2]. As a complex and potentially traumatic life event, perinatal loss not only causes great psychological trauma to the pregnant woman and her family but also has an impact on the next pregnancy. Studies have shown that women who experience perinatal loss have an increased risk of adverse pregnancy outcomes, such as stillbirth, preterm labour, placental abruption and neonatal death, and more prominent problems, such as sleep disorders, anxiety, depression and post-traumatic stress disorder (PTSD), when they become pregnant again compared with the general population [3–5].

A previous study by the group found that the prevalence of sleep disorders in late pregnancy was as high as 58.49% in women with a history of perinatal loss [6]. Poor sleep quality is an independent predictor of suicidal ideation among pregnant women [7]. Those with poor subjective sleep quality were 2.85 times more likely to experience suicidal ideation when compared with women who had good sleep quality. Studies in some countries have reported that short sleep duration during pregnancy is associated with a higher risk of preterm birth [8], low birth weight and small for gestational age [9]. Late pregnancy is a critical stage of pregnancy, and the quality of sleep is not only related to the health of the pregnant women themselves but may also have an important impact on fetal development and pregnancy outcomes [10,11].

In recent years, the sleep experiences in subsequent pregnancies of women with a history of perinatal loss during late pregnancy have not been sufficiently reported. Existing studies have mostly used quantitative methods to assess sleep quality and lack in-depth exploration of the subjective experience in this population. The present study comprehensively explores the sleep experience in late pregnancy of women with a history of perinatal loss through qualitative research methods, providing a basis for clinical healthcare professionals to develop effective interventions to improve the sleep quality of women and promote the health of mothers and infants.

## Materials and methods

### Design

A qualitative design with individual semi-structured in-depth interviews was used.

### Participant recruitment

Purposive sampling was used to select pregnant women in their third trimester who visited the obstetrics department of Suzhou Municipal Hospital between October 2023 and February 2024 as interview participants. The inclusion criteria were as follows: women (1) with prior miscarriage, stillbirth or neonatal death; (2) aged ≥20 years and at gestational week ≥28; (3) with certain reading comprehension and language expression ability; and (4) who provided informed consent and voluntary participation in this study. The exclusion criteria included women (1) with a previous history of mental illness or cognitive disorders; (2) combined with severe heart, brain, lung and other organ insufficiency; and (3) diagnosed with sleep disorders before pregnancy.

### Sampling strategy and sample size

Interviewees were selected in compliance with the principle of maximum difference sampling, and factors such as age, literacy, gestational week and reason for previous loss were considered in the selection of the women with a history of perinatal loss who were undergoing subsequent pregnancy. The sample size was based on the principle of saturation of interview data and no new themes.

### Interview guide

Two researchers developed a preliminary interview guide before conducting interviews, which was based on the study objectives [12], literature review and third-trimester pregnant women’s physiological and psychological characteristics. To ensure the validity of the outline, the researcher consulted an expert panel and pre-interviewed four pregnant women who met the inclusion criteria. The finalised interview outline was as follows: (1) Can you talk about your current sleep situation? (2) Do you think there are any changes in your sleep in this pregnancy compared with previous pregnancies? (3) What do you think are the factors affecting your sleep? (4) Have you taken any steps to improve your sleep, and can you be more specific? (5) What kind of help would you like the medical staff to give you?

### Data collection

Face-to-face interviews were conducted in a quiet and private conference room to protect the privacy of the interviewees and reduce their concerns. Before the interview, the researcher explained the purpose, significance and methodology of this study to the interviewees and obtained their written informed consent. Demographic data (age, education level, obstetric history) were collected and sleep quality was assessed using the Pittsburgh Sleep Quality Index (PSQI) [13] prior to the interview.

We applied Liu and Tang’s Chinese version to evaluate the sleep quality in the past month. The scale includes 19 self-assessment items and 5 other-assessment items, and the scoring items are the top 18 items in the self-assessment items. Scoring items contain 7 factors, each factor 0–3 scores, total score range 0–21 scores, the higher the score, the worse the sleep quality [14].PSQI total score greater than 7 scores for poor sleep quality.

All interviews were conducted by a lead researcher with training in qualitative methods and maternal and child health. A second researcher was present during each session to assist with observation, note-taking, and recording of participants’ facial expressions, body language, and emotional changes. Each interview lasted approximately 30–45 minutes and was audio-recorded with the participant’s permission.

### Data analysis

The researcher converted the audio recordings into text data and imported them into NVivo 11.0 (QSR International, Melbourne, Australia) within 24 hours after the interviews. The content analysis method [15] was used to analyse the interview data, in which two researchers independently analysed, coded, summarised and refined the data.

The analysis followed these steps: (1) converted the audio recordings into textual materials; (2) read the materials carefully; (3) extracted relevant information; (4) coded the recurring information; (5) refined the themes from coded information; (6) recorded the themes accurately to avoid omissions; and (7) returned to the interviewee for confirmation.

### Rigor and trustworthiness

To ensure the rigor and trustworthiness of the qualitative findings, several strategies were employed. The lead interviewer, a researcher trained in qualitative methods and maternal and child health, conducted all interviews, while a second researcher assisted with observation and note-taking. Prolonged engagement in the 30–45 minute interviews fostered rapport and yielded rich data. An audit trail documenting all research decisions ensured transparency and dependability. During analysis, two researchers independently coded the transcripts and resolved discrepancies through consensus, with the resulting themes reviewed by a third researcher with extensive qualitative experience. Participants were invited to review their transcribed interviews (member checking) to confirm accuracy. Additionally, the lead interviewer maintained a reflexive journal to document personal assumptions and potential biases. Although the research team shares a common background in maternal and child health nursing, independent coding, comparative discussions, and peer review by a senior qualitative researcher served as investigator triangulation to enhance credibility and confirmability.

### Ethics approval and consent to participate

This study was conducted in accordance with the Declaration of Helsinki and approved by the Ethics Committee of Suzhou Municipal Hospital (Approval No. K-2023–052-K01). Written informed consent was obtained from all participants. The study adhered to the principles of voluntary participation and confidentiality. Formal interviews commenced only after participants provided written informed consent, which included permission for the use of anonymized responses and direct quotations in publications. To ensure participant anonymity, all participants were assigned unique identification codes (e.g., A1, A2) to replace personal identifiers in interview transcripts and analyses. Any incidental references to third parties during the interviews were edited prior to data analysis. Access to the raw data was restricted solely to the research team.The ethics approval did not permit public sharing of full interview transcripts; however, access may be considered by the ethics committee upon reasonable request for legitimate research purposes.

## Results

A total of 18 participants were included in this study, aged 26–40 (32.67 ± 4.47) years and at gestational week 28–39 (32.20 ± 3.20). Previous causes of loss included miscarriages (n = 13), stillbirths (n = 3) and neonatal death (n = 2). The PSQI global scores ranged from 4 to 15, with a mean of 9.83 ± 3.24 and a median of 10 (IQR: 8–13). Using a cutoff of > 7 to define poor sleep quality, 14 participants (77.8%) were classified as having poor sleep quality. Participants were represented by A1–A18, and the general information is detailed in Table 1.

**Table 1:** Demographic characteristics of the participants (N = 18).

Interviewee	Age (years)	Educational Level	Employment Status	Previous loss type	Gestational weeks	Reasons for loss of the past	PSQI score
A1	30	College degree	Employed	G3P1	28	abortion	15
A2	26	College degree	Employed	G2P0	29	abortion	11
A3	33	High school	Employed	G2P0	37	stillbirth	14
A4	30	College degree	Employed	G2P0	33	abortion	5
A5	31	High school	Employed	G2P0	39	stillbirth	4
A6	29	Less than high school	Employed	G3P1	29	abortion	8
A7	33	Master’degree	Employed	G3P0	30	abortion	13
A8	39	College degree	Unemployed	G2P0	32	abortion	7
A9	33	College degree	Employed	G2P0	31	abortion	9
A10	37	High school	Unemployed	G3P1	35	neonatal death	13
A11	26	College degree	Employed	G2P0	33	abortion	10
A12	32	College degree	Employed	G2P0	34	abortion	8
A13	30	Master’degree	Employed	G3P1	34	neonatal death	10
A14	40	College degree	Employed	G5P2	28	abortion	6
A15	39	High school	Employed	G2P0	31	stillbirth	14
A16	36	Less than high school	Unemployed	G2P0	34	abortion	10
A17	35	College degree	Employed	G3P1	37	abortion	8
A18	37	High school	Employed	G3P1	34	abortion	12

Note: G, gravida (number of pregnancies); P, para (number of live births). PSQI, Pittsburgh Sleep Quality Index. Total score > 7 indicates poor sleep quality.

The researcher coded and refined the interview data, and finally summarised three themes: severe sleep disruption; diversified factors affecting sleep quality (distressing pregnancy symptoms, overloaded psychological load, behavioral and lifestyle factors, weak support from family and friends, and insufficient support from healthcare providers); and difficulties in self-coping with sleep problems.

### Theme1: Severe sleep disruption

During the interviews, most of the interviewees reflected that their sleep quality was poor during late pregnancy. They mainly suffered from insomnia, difficulty in falling asleep, increased number of awakenings during the night and even difficulty in falling asleep for the whole night at times.

*“My sleep quality was fine before pregnancy, but with this baby I started to suffer from frequent insomnia, and I could be easily awakened during the night, and I didn’t dare to take any medication, so I feel very irritable and I am also afraid that poor sleep will affect the baby.”(A2)*

*“I’m also very sleepy, but I just can‘t fall asleep. In short, it’s not an easy task to quickly enter a sleep state.”(A7)*

### Theme2: Diverse factors affecting sleep quality

Several factors influencing sleep were identified, including psychological burden, physiological symptoms, behavioral patterns, and support systems. Notably, although the interview guide specifically explored support from healthcare workers, participants consistently and spontaneously raised the issue of support from family and friends as a critical determinant of their sleep quality. These themes emerged naturally during the interviews without prompting from the interviewer.

**Psychological overload.** Previous experience of perinatal loss can cause pregnant women to be full of uncertainty about their current pregnancy, worrying about the health of the foetus and fearing another adverse pregnancy outcome. These psychological stresses can have a serious negative impact on the quality of sleep of pregnant women.

*“My baby has a heart defect. The doctors said we should prepare for the worst. At night I can’t stop thinking I’ll lose my child – the tears just come, and sleep won’t come either.”(A1)*

*“I had a miscarriage and underwent various tests before this pregnancy, ultimately deciding to proceed with IVF. There are a lot of unknown risks, I would often think about it at night, and I always have insomnia.”(A7)*

*“I am in advanced pregnancy, this is my last chance, I am worried about the baby’s health. With my due date approaching, the fear of history repeating itself is overwhelming. This constant worry follows me day and night – I can’t rest, can’t escape these terrifying thoughts.”(A10)*

**Disturbance by pregnancy symptoms.** Late pregnancy is the stage when the functional load of various organs of pregnant women reaches the highest level and the body shape changes significantly. Pregnant women are prone to pain, tiredness, dyspnea, frequent urination and other uncomfortable symptoms. Somatic symptom distress is a critical factor impairing sleep quality in women with a history of perinatal loss during subsequent pregnancy.

*“I have high blood pressure, and the doctor told me to take more rest, and it’s uncomfortable to lie down for a long time, and now the whole of the daytime and nighttime sleep has been reversed.” (A4)*

*“I have to change my position from time to time when I sleep at night, and I don’t sleep very well.” (A6)*

*“Fetal movements are very frequent, the baby is always kicking my stomach and seems to be in a sleepy state.”(A12)*

*“The belly is getting bigger and bigger, it’s not very convenient to turn over or do something, and sometimes I have to go to the toilet at night, and my sleep is interrupted.” (A8)*

**Behavioral and lifestyle factors.** Four pregnant women stated that they would use electronic products for leisure and entertainment activities at night, and had sleep procrastination behaviours. Others mentioned their irregular work and rest schedules, which would have an impact on the quality of their sleep.

*“If you can’t resist the temptation, such as getting addicted to reading cell phones, watching movies or watching certain videos, you’ll throw away the common sense that it is not good for the body to stay up late, and also get addicted to chatting with other people. And when you get addicted to chatting with others, you sleep later. ”(A18)*

*“Eating a little too much at night is indigestible, and the top of my stomach is uncomfortable, so I don’t sleep very well.”(A3)*

*“When I sleep a lot during the day, I don’t sleep much at night, and the next day, I’m sleepy during the day, it’s just a vicious circle.”(A5)*

**Weak support from family and friends.** Respondents felt that when discussing their sleep problems with family and friends, they did not receive sufficient understanding and recognition to obtain effective advice.

*“My husband dismisses my concerns as exaggerated. I have no one to share my worries with, and often feel sad at night, which affects my sleep. I would just play with my phone and listen to some light music, but the effect was also very limited. ”(A2)*

*“I can’t tell my husband when I’m not feeling well, and when I do, he says I’m making a big deal out of it. There’s no one to talk to about what’s on my mind, and sometimes when I lie down at night, I feel a little sad and can’t sleep well.”(A9)*

*“My friend told me that it’s normal to have trouble sleeping at this stage, and it’s best not to take medication, as it’s not good for the baby.” (A15)*

Despite the prevailing lack of support, one participant did report receiving understanding and encouragement from their families.

*“Whenever I have trouble falling asleep, my husband will massage me to help me relax. This makes me no longer feel lonely.” (A17)*

**Insufficient support from healthcare providers.** In addition to the support from family and friends, several participants also expressed that healthcare professionals offered limited guidance regarding sleep management during late pregnancy. While some received brief reassurance or pharmacological interventions, they often felt that the support was insufficient or that medication-related concerns remained unaddressed.

*“Doctors and nurses are very busy, they can‘t do everything, sometimes they just ask if I slept well.” (A11)*

*“The nurse told me to relax and not to be anxious, or I would sleep even worse. Once I couldn’t sleep and I took Valium [medication], I was actually worried that the side effects would be harmful to the baby.” (A14)*

### Theme3: Difficulty in self-coping with sleep problems

Three women with a history of perinatal loss undergoing subsequent pregnancy reported adopting no sleep-promoting measures despite experiencing sleep disturbances.

*“It is normal to have a bad sleep now, and there is no better way to improve it, and it will be fine if you stick with it for a while longer after you give birth to the baby.” (A2)*

*“There’s no real solution – when sleepless, I simply lie there with closed eyes, grabbing what little shallow sleep exhaustion forces upon me.” (A13)*

In addition, four pregnant women took measures they thought might be effective, but the results were unsatisfactory, and they lacked the knowledge and ability to cope themselves.

*“When I can’t sleep, I just listen to some sleep-aiding music or play with my cell phone for a while, which doesn’t have much effect, and sometimes I still can’t sleep.” (A14)*

*“It’s especially hard to sleep every night, sometimes it’s more comfortable to use a thick quilt placed on the side of the bed and lean against it, that’s all I can do.” (A15)*

## Discussion

The results of the qualitative interviews showed that women with a history of perinatal loss have poor sleep quality and poor sleep experience in subsequent late pregnancy and that their sleep experience needs to be improved urgently. During late pregnancy, many women face sleep difficulties and fatigue due to changes in body size and increased cardiac load [16]. Pregnant women with a history of adverse maternal outcomes often require more complex treatments and more frequent prenatal visits, which further adds to their physical burden. In addition to pregnancy-induced physical exhaustion, this population also has to cope with the stress associated with high-risk pregnancies [17], all of which may exacerbate sleep problems by aggravating the psychological stress response of pregnant women.

During the interviews, some interviewees mentioned that they were concerned that insomnia would affect their unborn children, which indicates that some pregnant women place a certain degree of importance and concern on the quality of sleep during pregnancy. However, in late pregnancy, medical staff and family members are more concerned about the presence of pregnancy complications and the ability of the pregnant women to deliver their babies successfully, and sleep problems are not seen as an urgent or priority issue. Therefore, to improve the sleep experiences of women with a history of perinatal loss during subsequent pregnancies, healthcare providers should gain an in-depth understanding of their sleep needs, systematically analyse and understand the reasons for the occurrence of sleep problems and incorporate sleep quality into routine follow-up. Furthermore, they should assess pregnant women’s sleep by using sleep-related scales and sleep diaries, and provide the necessary assistance to optimise their sleep experience in late pregnancy.

The results of this study showed that psychological overload and late pregnancy somatic symptoms are important factors affecting the quality of sleep in women with a history of perinatal loss. As a stressful event, perinatal loss causes persistent psychological trauma in pregnant women [18], leading to excessive tension, worry and sensitivity during subsequent pregnancy. For women with a history of perinatal loss who are pregnant again, the late stage of pregnancy not only involves a heavy physiological load but also a greater psychological pressure. One survey shows that PTSD rates following perinatal loss are up to seven times the rates among mothers of living infants [19]. Negative emotions such as worry or anxiety before bedtime can lead to sympathetic nervous system excitation, elevated cortisol levels and altered melatonin levels, resulting in circadian rhythm disruption and affecting the quality of sleep in pregnant women [20]. Analysis of the causes revealed that as the weeks of pregnancy increase, the uterus continues to grow and exerts increasing pressure on the bladder, which leads to an increase in the frequency of nocturnal urination and awakenings in pregnant women [21]. Lower back pain in some pregnant women may be related to relaxation of pelvic ligaments and intervertebral nodes and ligaments by relaxin secreted by the placenta, and musculoskeletal discomfort associated with fetal movement and musculoskeletal changes during pregnancy can also lead to sleep fragmentation. Multiple symptoms synergistically interact, significantly impairing sleep quality in women with a history of perinatal loss during subsequent pregnancy [22]. This suggests that medical personnel should strengthen the symptom management of subsequent late pregnancy for women experiencing perinatal loss, identify the somatic symptoms of the women in a timely manner and encourage them to report the symptoms and sleep status on their own. Moreover, the risk perception caused by sleep disorders and the distress of somatic discomfort should be improved, and targeted and effective strategies should be adopted, such as instructing pregnant women to adopt the left lateral position to alleviate and relieve the uterus’s pressure on the inferior vena cava and increase the venous reflux. Appropriate outdoor activities should be recommended to promote blood circulation, improve sleep and increase appetite, while drinking a lot of water before bedtime should be avoided to reduce the frequency of nighttime toilet visits, thereby enhancing the quality of nighttime sleep.

In addition, increased attention should be paid to the mental health of women with a history of perinatal loss during subsequent pregnancy. Health education should be strengthened to guide these women in managing emotions and stress effectively. Psychological elasticity plays an important role in stress coping. Individuals with higher psychological elasticity have a stronger ability to self-regulate and emotionally regulate in the face of adversity or stress, and are able to effectively alleviate anxiety and stress, thus improving sleep quality. Therefore, medical personnel can improve the psychological elasticity of pregnant women through a series of measures, such as emotional calming strategies [23] and positive thinking stress reduction training [24], to reduce pregnancy stress and promote psychological health, thereby improving sleep quality.

Poor sleep habits, irregular work routines, lack of sleep health awareness and serious sleep procrastination behaviours can affect the quality of sleep in women with a history of perinatal loss during subsequent pregnancy. On analysing the factors, the use of electronic products for leisure and recreational activities at night will clearly affect sleep, with the continuous light emitted by the devices interfering with the formation of circadian rhythms in pregnant women, leading to a misalignment of the individual’s perception of time, and, at the same time, inhibiting the production of melatonin, thus affecting sleep quality [25]. Furthermore, irregular work and rest schedules can disrupt the body’s biological clock, leading to unstable sleep quality, and excessive sleep during the day can lead to insomnia at night, forming a vicious cycle that affects sleep quality and quality of life [26]. It is suggested that nursing staff should guide pregnant women to create a good sleep atmosphere in advance, reduce the temptation of accessible networks, enhance their sleep health awareness and help them to establish a stable sleep schedule, such as regular sleep and wake-up times, as well as avoiding long naps. Continuing health education groups have been formed to provide pregnant women with online guidance and interactive learning through a variety of means, such as webcasting, microblogging platforms and multimedia, while a web-based follow-up platform has been established for regular follow-up visits.

The results of this study show that a weak support system is also an influential factor in the quality of sleep in women with a history of perinatal loss during subsequent pregnancy. The interviewees noted that when they attempted to discuss their sleep problems with family members or medical personnel, they often did not receive adequate understanding and recognition, nor did they receive substantial help or solutions. One participant also mentioned that her friend‘s advice regarding sleep was unhelpful, though this was not a prevalent theme across the sample. The lack of information on ways to improve sleep quality, the use of hypnotic medications and so on can prevent pregnant women from effectively dealing with their sleep problems, leading to decreased sleep quality. Family support refers to the positive and favourable assistance provided by family members in the emotional, psychological and daily life aspects, which can maintain harmonious family relationships and promote efficient solutions to various problems [27]. One study also found that comprehensive family-centred interventions can alleviate pregnant women’s sleep disorders and help improve their quality of life in late pregnancy and maintain a healthy lifestyle [28]. It is suggested that medical personnel can build a family-centred multifaceted support system, pay attention to sleep assessment and provide personalised sleep advice and medication management to help pregnant women who have experienced perinatal loss to effectively resolve their sleep problems, thereby promoting the health of the mother and infant. Moreover, spouses and family members of pregnant women are advised to learn the contents and forms of family support, provide emotional and behavioral support for pregnant women and promote positive communication and exchange to improve sleep problems.

Notably, one participant described receiving emotional and physical comfort from her husband during episodes of sleep difficulty, suggesting that positive partner support may serve as a protective factor against sleep disturbances. However, this was not commonly reported across the sample, which may reflect the heightened sensitivity and vulnerability of this population, or a genuine gap in available support. Additionally, while peer support has been shown in previous studies to improve emotional well-being and sleep quality in perinatal populations [29], this was not directly reported by participants in our study and warrants further investigation.

This study also found that some pregnant women lacked effective coping knowledge and coping skills when they experienced sleep problems. A self-management model involves individuals adopting specific behaviours to maintain and improve their health, monitor and manage their physical and psychological symptoms, and treat their illnesses consistently [30]. Pregnant women experiencing insomnia can acquire the skills and methods of disease management, which can improve their confidence in coping with sleep problems. In addition, healthcare professionals can assist pregnant women in finding effective treatments to improve their sleep quality. Recent studies confirm that wearable technology can continuously and autonomously track adult sleep patterns while delivering quantifiable sleep parameters via digital interfaces [31,32]. It is suggested that caregivers can instruct pregnant women to wear portable health monitoring devices, such as smart bracelets, to track and monitor their health and living conditions, and to obtain more accurate information to optimise sleep patterns and provide more effective interventions, as well as to improve self-management.

Traditional Chinese medicine (TCM) therapies, such as footbaths and acupressure, may offer alternative approaches to managing insomnia during pregnancy [33], though their effectiveness in this specific population warrants further investigation.

### Limitations

This study used semi-structured interviews to gain insights into the late pregnancy sleep experiences of women with a history of perinatal loss during subsequent pregnancy, laying the foundation for more effective clinical interventions. However, this study has certain limitations. First, the sample was limited to a specific geographic area and healthcare organisation, limiting the generalisability of the findings. Second, there were limitations in the sample selection process. Only pregnant women in late pregnancy were recruited for this study, which did not allow for a comprehensive understanding of the population’s sleep experiences throughout the pregnancy period.

## Conclusion

In this study, 18 women experiencing perinatal loss were interviewed face to face about their sleep experience in late pregnancy. Three main themes were identified: serious sleep disruption; diverse factors affecting sleep quality (pregnancy symptoms, psychological overload, disturbed sleep patterns, and weak family and social support); and difficulties in self-coping with sleep problems. Healthcare professionals should improve the sleep quality of women experiencing perinatal loss during subsequent pregnancy through a series of measures. This could include timely recognition of somatic symptoms of pregnancy discomfort, attention to the women’s mental health status, change of irrational cognition, enhancement of sleep health awareness, establishment of diversified support and improvement of self-coping ability.

## Supporting information

S1 Table All anonymized quotations cited in the manuscript, organized by themes. (DOCX)
