# Supplementary material for: A qualitative study on sleep experiences in late pregnancy among women with a history of perinatal loss
Source: PLoS One. 2026 Aug 19;21(8):e0354895. doi: 10.1371/journal.pone.0354895 (PMC13489473; doi:10.1371/journal.pone.0354895)
Supplement: Supplementary file 1 [file pone.0354895.s001.docx]

**S1 Table. All anonymized quotations cited in the manuscript, organized by themes. Full interview transcripts are not publicly available due to ethical restrictions.**

| **Theme** | **Key illustrative quotes** |
| --- | --- |
| **Theme1: Severe sleep disruption** | A2: “My sleep quality was fine before pregnancy, but with this baby I started to suffer from frequent insomnia, and I could be easily awakened during the night, and I didn’t dare to take any medication, so I feel very irritable and I am also afraid that poor sleep will affect the baby.” |
|  | A7: “I’m also very sleepy, but I just can‘t fall asleep. In short, it’s not an easy task to quickly enter a sleep state.” |
| **Theme2: Diverse factors affecting sleep quality**  **－Psychological overload** | A1: “My baby has a heart defect. The doctors said we should prepare for the worst. At night I can’t stop thinking I’ll lose my child – the tears just come, and sleep won’t come either.” |
|  | A7: “I had a miscarriage and underwent various tests before this pregnancy, ultimately deciding to proceed with IVF. There are a lot of unknown risks, I would often think about it at night, and I always have insomnia.” |
|  | A10: “I am in advanced pregnancy, this is my last chance, I am worried about the baby’s health. With my due date approaching, the fear of history repeating itself is overwhelming. This constant worry follows me day and night – I can’t rest, can’t escape these terrifying thoughts.” |
| **Theme2: Diverse factors affecting sleep quality**  **－Disturbance by pregnancy symptoms** | A4: “I have high blood pressure, and the doctor told me to take more rest, and it’s uncomfortable to lie down for a long time, and now the whole of the daytime and nighttime sleep has been reversed.” |
|  | A6: “I have to change my position from time to time when I sleep at night, and I don’t sleep very well.” |
|  | A12: “Fetal movements are very frequent, the baby is always kicking my stomach and seems to be in a sleepy state.” |
|  | A8: “The belly is getting bigger and bigger, it’s not very convenient to turn over or do something, and sometimes I have to go to the toilet at night, and my sleep is interrupted.” |
| **Theme2: Diverse factors affecting sleep quality**  **－Behavioral and lifestyle factors** | A18: “If you can’t resist the temptation, such as getting addicted to reading cell phones, watching movies or watching certain videos, you’ll throw away the common sense that it is not good for the body to stay up late, and also get addicted to chatting with other people. And when you get addicted to chatting with others, you sleep later. ” |
|  | A3: “Eating a little too much at night is indigestible, and the top of my stomach is uncomfortable, so I don’t sleep very well.” |
|  | A5: “When I sleep a lot during the day, I don’t sleep much at night, and the next day, I’m sleepy during the day, it’s just a vicious circle.” |
| **Theme2: Diverse factors affecting sleep quality**  **－Weak support from family and friends** | A2: “My husband dismisses my concerns as exaggerated. I have no one to share my worries with, and often feel sad at night, which affects my sleep. I would just play with my phone and listen to some light music, but the effect was also very limited. ” |
|  | A9: “I can’t tell my husband when I’m not feeling well, and when I do, he says I’m making a big deal out of it. There’s no one to talk to about what’s on my mind, and sometimes when I lie down at night, I feel a little sad and can’t sleep well.” |
|  | A15: “My friend told me that it’s normal to have trouble sleeping at this stage, and it’s best not to take medication, as it’s not good for the baby.” |
|  | A17: “Whenever I have trouble falling asleep, my husband will massage me to help me relax. This makes me no longer feel lonely.” |
| **Theme2: Diverse factors affecting sleep quality**  **－Insufficient support from healthcare providers** | A11: “Doctors and nurses are very busy, they can‘t do everything, sometimes they just ask if I slept well.” |
|  | A14: “The nurse told me to relax and not to be anxious, or I would sleep even worse. Once I couldn’t sleep and I took Valium [medication], I was actually worried that the side effects would be harmful to the baby.” |
| **Theme3: Difficulty in self-coping with sleep problems** | A2: “It is normal to have a bad sleep now, and there is no better way to improve it, and it will be fine if you stick with it for a while longer after you give birth to the baby.” |
|  | A13: “There’s no real solution – when sleepless, I simply lie there with closed eyes, grabbing what little shallow sleep exhaustion forces upon me.” |
|  | A14: “When I can’t sleep, I just listen to some sleep-aiding music or play with my cell phone for a while, which doesn’t have much effect, and sometimes I still can’t sleep.” |
|  | A15: “It’s especially hard to sleep every night, sometimes it’s more comfortable to use a thick quilt placed on the side of the bed and lean against it, that’s all I can do.” |
